# Trust-based fault detection and robust fault-tolerant control of uncertain cyber-physical systems against time-delay injection attacks^☆^

**DOI:** 10.1016/j.heliyon.2021.e07294

**Published:** 2021-06-15

**Authors:** Salman Baromand, Amirreza Zaman, Lyudmila Mihaylova

**Affiliations:** a Department of Electrical Engineering, Fasa University, Fasa, Iran; b Control Engineering Group, Department of Computer Science, Electrical and Space Engineering, Luleå University of Technology, Luleå, Sweden; c Department of Automatic Control and Systems Engineering, University of Sheffield, Sheffield, UK

**Keywords:** Correlation analysis, Cyberattacks, Kullback-Leibler divergence, Linear matrix inequalities (LMIs), Robust control, Uncertain systems, Unknown time-delay attacks

## Abstract

Control systems need to be able to operate under uncertainty and especially under attacks. To address such challenges, this paper formulates the solution of robust control for uncertain systems under time-varying and unknown time-delay attacks in cyber-physical systems (CPSs). A novel control method able to deal with thwart time-delay attacks on closed-loop control systems is proposed. Using a descriptor model and an appropriate Lyapunov functional, sufficient conditions for closed-loop stability are derived based on linear matrix inequalities (LMIs). A design procedure is proposed to obtain an optimal state feedback control gain such that the uncertain system can be resistant under an injection time-delay attack with variable delay. Furthermore, various fault detection frameworks are proposed by following the dynamics of the measured data at the system's input and output using statistical analysis such as correlation analysis and K-L (Kullback-Leibler) divergence criteria to detect attack's existence and to prevent possible instability. Finally, an example is provided to evaluate the proposed design method's effectiveness.

## 1 Introduction

During the last few years, uncertain systems have been widely encountered because of the environmental changes, systems' failures, and disturbances [1], [2]. These systems have been widely used in the application of electronic circuits, power systems, spring damping systems, and mechanical systems [3], [4], [5]. Since the industrial application of cyber-physical uncertain systems increases, stealthy cyber-attacks may occur to prevent systems' productivity and degrade systems' performances.

One important factor that can cause instability in most practical systems is time-delay effects. These effects in nonlinear systems can be the main reason for weaknesses of control approaches in most cases [6], [7]. Therefore, several robust and adaptive strategies have been done to control uncertain and nonlinear systems under time-delay effects with the assumption of parametric uncertainties. Therefore, several robust and adaptive strategies have been done to control uncertain and nonlinear systems under time-delay effects with the assumption of parametric uncertainties [8], [9], [10], [11], [12], [13], such as approaches with backstepping and dynamic surface designs [14], [15]. In [16], a set of network-based uncertain systems is introduced by modeling these problems with event-triggered robust filtering. A recent event-triggered control proposition is stated in [17] to synchronize a switched delayed neural network. When it comes to decentralized nets, in [18], the event-triggered filtering for a decentralized network interconnecting nonlinear system is developed. Also, some event-triggered methods using a periodic sampled-data control and with fuzzy systems are introduced in [19], [20], [21], [22], [23]. In [24], the networked control of uncertain nonlinear systems is investigated with an adaptive event-triggered strategy under unknown time-delay conditions.

Even though various studies have been done regarding robust and adaptive control of uncertain systems, a few approaches are related to designing the robust and adaptive control strategies of uncertain CPSs with time delays and considering cyber components. Besides, because of the unavailability of error surfaces of the measured and sent state information by sensors in CPSs, which various attackers could alter these surfaces, previous approaches [11], [12], [13], [14], [15], [16], [17], [18], [19], [20], [21], [22], [23], [24] cannot be applied to prevent cyberattacks in uncertain CPSs. Thus, the problem of designing control methods to resist uncertain time-delayed CPSs against different attack strategies is still an interesting subject to follow and consider.

Some approaches have been devoted to state the importance of the security issue of CPSs [25], [26], [27], [28], [29]. Control problems in these systems are categorized into three groups based on different cyber-attack scenarios; denial-of-service (DoS) attacks [30], [31], [32], [33], deception attacks [34], [35], [36], [37], [38], and replay attacks [39], [40], [41]. The latest studies regarding security control of CPSs against cyberattacks are as follows. In [42], the issue of designing event-based security control for uncertain state-dependent systems with the existence of hybrid attacks is proposed. Besides, the finite-time $H_{\infty }$ filtering method for networked state-dependent uncertain systems under multiple attacks (DoS, deception, and replay attacks) by considering the event-triggered approach is investigated in [43]. Another control strategy of nonlinear time-delayed CPSs under unspecific deception attacks is developed in [44], in which an adaptive, resilient, dynamic surface control using the neural-network scheme is proposed for deception attacks on both sensor and actuator sides.

One kind of malicious attack that can cause trouble in operating uncertain systems is a time-delay attack. A time-delay attack on a control system is the reason for adversaries that fundamentally add time delays into such systems and potentially forcing them to instability and crash. A recent approach has been made regarding time-delay attacks [45]. First, it is shown that cryptographic methods against these attacks would be useless in detecting cyber components. A cryptography-free time-delay attack recovery (CF-TDR) communication protocol is developed to identify failures and recover from these attacks' destructive effects.

Previous designed robust control approaches against time delays considered the existence of time-independent delays with known values. There is still no approach to investigating time-delay attacks with unknown and time-varying values on uncertain systems with a robust approach to detect these attacks and recover the system's performance. Various protection-based methods against data injection attacks have been developed lately. Most of them involve using protected measurement data or using estimated system data. However, if the attackers can infiltrate security systems and manipulate metering data, the control system will be compromised. On the other hand, these methods may not detect contaminated data with a statistical distribution similar to the previously measured safe data.

It is well known that a robust controller can often maintain states of a system bounded against various types of uncertainties, which can be the modeling uncertainties or environmental uncertainties. On the other hand, if unknown and time-varying delays are injected into the system's performance by an intruder, formerly applied robust control approaches cannot be further beneficial. Consequently, the uncertain system's stability under unknown and time-varying delays will be enhanced, and hence, the states of the system will remain bounded under various time delays. Besides, it is necessary to detect the delay's occurrence to implement other safety protocols. The main contributions are itemized as follows:

1. We allocate a robust controller that attenuates and partially eliminates the harmful effects of delayed contaminated data injection on system stability. Besides, to show the proposed approach's efficiency, the amount of delay is assumed to be randomly selected. Therefore, the designed feedback controller will prevent system instability based on random time delays.
2. We define uncertainties in the system as unknown values with the norm-bounded feature. Using a descriptor model representation and an appropriate Lyapunov functional, we formulate sufficient conditions for closed-loop stability based on the linear matrix inequalities (LMIs).
3. We present the online fault detection framework by following the dynamics of the measured data at the system's input and output to prevent the operating system from going into the faulty phase as quickly as possible. Attack detection methods are proposed and compared using statistical analysis such as correlation analysis and K-L divergence attack detection criteria.
4. Simulation results verify that the considered uncertain system will be resilient against malicious time-delay attacks. Therefore, the trustworthiness of the system's performance will be enhanced over time by applying the developed robust control protocol. Additionally, using the provided fault detection strategies, stealthy time-delay attacks can be detected at the time of their occurrence.

This paper's remainder is as follows: In Section 2, the uncertain control system is analyzed under the time-delay attack, and then, the robust $H_{\infty }$ delay-independent controller is proposed to overcome instability conditions in the system. In Section 3, various statistical attack detection methods are reviewed to detect faults in the system under the time-delay attacker's existence. Eventually, numerical simulations and concluding sections are presented in Sections 4 and 5, respectively.

## 2 Time-delay attack analysis for uncertain systems

### 2.1 Problem statement

Real-life processes in a smart fashion such as smart industrial systems can be modeled as nonlinear systems. Assume the nonlinear system given by the below state-space equations

(1) $$\overset{˙}{x}\left(t\right)=\overset{˜}{f}\left(x\right)+\overset{˜}{B}u(t),\;y(t)=Cx(t),$$

where $x\in R^{n}$ and $u\in R^{m}$ are the state and the control input vectors, respectively. Furthermore, $\overset{˜}{B}$ is the input matrix, $\overset{˜}{f}\left(x\right)$ is the nonlinear function of system states, and C is the output matrix of the system. Suppose the obtained linearized model around its equilibrium point is controllable and also has the state-space matrices (A,B). So, $\overset{˜}{f}\left(x\right)$ and $\overset{˜}{B}$ can be decomposed as

(2) $$\left\{ \begin{array}{c} \overset{˜}{f}\left(x\right)=\left(A+\Delta A\right)x(t),\\ \overset{˜}{B}=\;B+\Delta B, \end{array}\right.$$

where Δ*A*, Δ*B* are model uncertainties and *A*, *B*, Δ*A*, and Δ*B* have applicable dimensions. Thus, the model of continuous-time uncertain system (1) (or approximation of system in a region of interest by an LTI system), can be expressed as following uncertain state space equations

(3) $$\overset{˙}{x}\left(t\right)=\left(A+\Delta A\right)x\left(t\right)+\left(B+\Delta B\right)u(t),y(t)=Cx(t).$$

It is assumed that the attacker tries to inject a time-delay attack to force the system to be unstable or have abnormal operations. We can consider the given uncertain system (4) with an augmented time-delay attack as:

(4) $$\overset{˙}{x}\left(t\right)=\left(A+\Delta A\right)x\left(t\right)+\left(B+\Delta B\right)u(t)+\sum_{i=1}^{k}{\overset{˜}{D}}_{i}\overset{˙}{x}\left(t-g_{i}(t)\right)+\sum_{i=1}^{k}H_{i}x\left(t-g_{i}(t)\right),$$

where ${\overset{˜}{D}}_{i}\;\text{and}\;H_{i}$ are the system matrices with appropriate dimensions and also $\sum_{i=1}^{k}{\overset{˜}{D}}_{i}\overset{˙}{x}\left(t-g_{i}(t)\right)+\sum_{i=0}^{k}H_{i}x\left(t-g_{i}(t)\right)$ is assumed as a delay attack strategy, with $g_{i}(t)\geq 0$. It should be noted that the delay term $g_{i}(t)$ is assumed to be time-varying to obtain more general results in the paper for a worst-case scenario and to show the effectiveness of the proposed robust control solution with any random values of $g_{i}(t)$ in further analysis.

**Figure 1:**
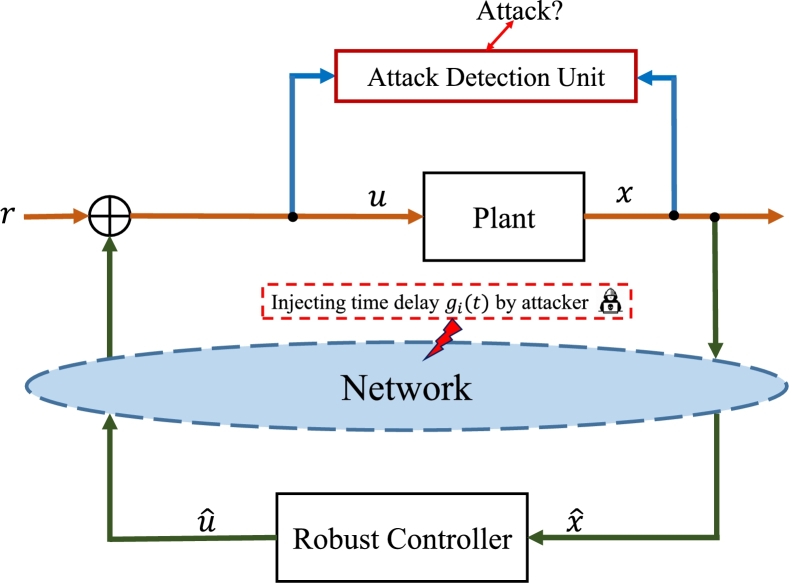
The structure of the CPS with the proposed robust controller and attack detection unit under unknown time-delay attacks.

The purpose of the control solution protocol is to frame a delay-independent robust $H_{\infty }$ controller u(t)=Kx(t), which assures the robust stability of the system under delay attacks for maximum and unknown delay $g_{i}(t)\geq 0$. The structure of robust security control for the networked control system with time-delay attacks is illustrated in Fig. 1.

### 2.2 Robust $H_{\infty }$ controller design for closed-loop uncertain CPSs

With the proposed robust $H_{\infty }$ control law u(t)=Kx(t), we have a closed-loop system as

(5) $$\overset{˙}{x}\left(t\right)={\overset{˜}{A}}_{0}x(t)+\sum_{i=1}^{k}{\overset{˜}{D}}_{i}\overset{˙}{x}\left(t-g_{i}(t)\right)+\sum_{i=1}^{k}H_{i}x\left(t-g_{i}(t)\right),$$

where *K* is the robust controller feedback gain and

(6) $${\overset{˜}{A}}_{0}=A_{0}+\Delta A_{0},$$

$$\left(A_{0}=A+BK,\;\;\;\;\;\Delta A_{0}=\Delta A+\Delta BK\right)$$

and

(7) $${\overset{˜}{D}}_{i}={\bar{D}}_{i}+\Delta \bar{D_{i},}$$

where $\bar{D}$_*i*_ and $\Delta \bar{D_{i}}$ are specific fixed-valued real matrices of applicable dimensions. Moreover, the possible system's uncertainties are described by the given equations

(8) $$\left[\begin{array}{cc} \Delta A\; & \Delta B \end{array}\right]=D_{0}F_{0}(x,t)\left[\begin{array}{cc} E_{a}\; & E_{b} \end{array}\right],$$

(9) $$\Delta \bar{D_{i}}=D_{i}F_{i}(x,t){\bar{E}}_{i},$$

where *D*_0_*, E*_*a*_*, E*_*b*_*, D*_*i*_*,* and ${\bar{E}}_{i}$ are fixed-valued real matrices of applicable dimensions. Besides, $F_{i}(x,t)$ and $F_{0}(x,t)$ are anonymous real-valued time-varying matrices with Lebesgue measurable items complying the given bounds

(10) $$F_{i}^{T}\left(x,t\right)F_{i}\left(x,t\right)\leq I,$$

(11) $$F_{0}^{T}\left(x,t\right)F_{0}\left(x,t\right)\leq I,\;\;\;\;\;\forall t.$$

**Assumption A1.** In deriving formulas, it is assumed that:

(12) $$\sum_{i=1}^{k}\left|{\overset{˜}{D}}_{i}\right|<1,$$

where |.| is any matrix norm. With the above assumption, both stability conditions associated with continuous and continuously differentiable initial functions are equivalent. To derive stability conditions, the following lemmas are essential to mention.

Lemma 1 **[46], [47]**

*For any* $z,\;y\in R^{n}$ *and any positive definite matrix* $X\in R^{n\times n}$*,*

(13) $$-2z^{T}\;y\leq z^{T}X^{-1}z+y^{T}Xy.$$

Lemma 2 **[46], [47]**

*Let A, D, E, and F be real matrices of appropriate dimensions with* ‖F‖≤I*. Accordingly, it can be concluded that:*

*For any scalar* ε>0*,*

(14) $$DFE+E^{T}F^{T}D^{T}\leq \varepsilon^{-1}DD^{T}+\varepsilon E^{T}E,$$

*for any matrix* H>0 *and scalar* ε>0*, which applies to the inequality* $\varepsilon I-EHE^{T}>0$*,*

(15) $$\left(A+DFE\right)H{\left(A+DFE\right)}^{T}\leq AHA^{T}+AHE^{T}{\left(\varepsilon I-EHE^{T}\right)}^{-1}EHA^{T}+\varepsilon DD^{T},$$

*for any matrix* H>0 *and scalar* >0*, which applies to the inequality* $H-\varepsilon DD^{T}>0$*,*

(16) $${\left(A+DFE\right)}^{T}H^{-1}\left(A+DFE\right)\leq A^{T}{\left(H-\varepsilon DD^{T}\right)}^{-1}A+\varepsilon^{-1}E^{T}E.$$

### 2.3 Delay-independent stability for an uncertain system with time–delay attack

To find delay-independent stability condition, the descriptor representation of the system is given as follows

(17) $$\overset{˙}{x}\left(t\right)=y\left(t\right),$$

(18) $$y\left(t\right)={\overset{˜}{A}}_{0}x\left(t\right)+\sum_{i=1}^{k}{\overset{˜}{D}}_{i}y\left(t-g_{i}(t)\right)+\sum_{i=1}^{k}H_{i}x\left(t-g_{i}(t)\right).$$

By defining the Lyapunov-Krasovskii functional candidate as:

(19) $$V\left(t\right)=\left[\begin{array}{cc} x^{T}\left(t\right)\; & y^{T}\left(t\right) \end{array}\right]EP\left[\begin{array}{c} x\left(t\right)\\ y\left(t\right) \end{array}\right]+V_{1}+V_{2,}$$

where

$$E=\left[\begin{array}{cc} I\; & 0\\ 0\; & 0 \end{array}\right],\;\;\;P=\left[\begin{array}{cc} P_{1}\; & 0\\ P_{2}\; & P_{3} \end{array}\right],\;\;P_{1}=P_{1}^{T}>0,$$

and

(20) $$V_{1}=\sum_{i=1}^{k}\int_{t-g_{i}}^{t}y^{T}\left(s\right)Q_{i}y^{T}\left(s\right)ds,\;\;Q_{i}>0,$$

(21) $$V_{2}=\sum_{i=1}^{k}\int_{t-g_{i}}^{t}x^{T}\left(s\right)U_{i}x^{T}\left(s\right)ds,\;\;U_{i}>0,$$

further results from the Theorem 1 can be concluded.

Theorem 1 *Under assumption A1, the uncertain system* (3) *under the injection time-delay attack introduced in* (4) *is stable for all delay values* $g_{i}(t)>0,\;i=1,\ldots ,k$ *if there is a matrix* $X=\left[\begin{array}{cc} X_{1}\; & 0\\ X_{2}\; & X_{3} \end{array}\right]$*,* $X_{1}=X_{1}^{T}>0,\;X_{2}\;,\;X_{3},{\bar{Q}}_{i}={\bar{Q}}_{i}^{T}\;,\;{\bar{U}}_{i}={\bar{U}}_{i}^{T},\;i=1,\ldots ,k$ *that satisfies the following LMI*

(22) $$W=\left[\begin{array}{cccccc} {\overset{=}{\psi }}_{1}\; & {\bar{\theta }}_{1}\; & 0\; & {\bar{\theta }}_{2}\; & X^{T}vec\left\{I\right\}\; & X^{T}vec\left\{I\right\}\\ ⁎\; & {\bar{\theta }}_{3}\; & {\bar{\theta }}_{4}\; & 0\; & 0\; & 0\\ ⁎\; & ⁎\; & {\bar{\theta }}_{5}\; & 0\; & 0\; & 0\\ ⁎\; & ⁎\; & ⁎\; & {\bar{\theta }}_{6}\; & 0\; & 0\\ ⁎\; & ⁎\; & ⁎\; & ⁎\; & {\bar{\theta }}_{7}\; & 0\\ ⁎\; & ⁎\; & ⁎\; & ⁎\; & ⁎\; & {\bar{\theta }}_{8} \end{array}\right]<0,$$

*where*

(23) $${\overset{=}{\psi }}_{1}=\left[\begin{array}{cc} 0\; & 0\\ AX_{1}+BY\; & 0 \end{array}\right]+\left[\begin{array}{cc} 0\; & {\left(AX_{1}\right)}^{T}+{\left(BY\right)}^{T}\\ 0\; & 0 \end{array}\right]+\left[\begin{array}{cc} 0\; & I\\ 0\; & -I \end{array}\right]X\;\;\;\;\;\;+X^{T}\left[\begin{array}{cc} 0\; & 0\\ I\; & -I \end{array}\right]+\left[\begin{array}{cc} 0\; & 0\\ 0\; & 2\sum_{i=1}^{m}\xi_{i}^{-1}D_{i}D_{i}^{T} \end{array}\right]+\left[\begin{array}{cc} 0\; & 0\\ 0\; & \xi_{0}^{-1}D_{0}D_{0}^{T} \end{array}\right]\;\;\;\;\;+X^{T}\left[\begin{array}{cc} \sum_{i=0}^{m}\xi_{i}E_{i}^{T}E_{i}\; & 0\\ 0\; & 0 \end{array}\right]X.$$

*Additionally, other variables are defined as*

$${\bar{\theta }}_{1}=vec\left\{\left[\begin{array}{c} 0\\ {\bar{D}}_{i} \end{array}\right]\left({\bar{Q}}_{i}\right)\right\},\;{\bar{\theta }}_{2}=vec\;\left\{\left[\begin{array}{c} 0\\ H_{i} \end{array}\right]\left({\bar{U}}_{i}\right)\right\},{\bar{\theta }}_{3}=-diag\left({\bar{Q}}_{i}\right),{\bar{\theta }}_{4}=vec\left\{{\bar{E}}_{i}^{T}\right\},$$

$${\bar{\theta }}_{5}=-diag\left(\xi_{i}^{-1}\;I\right),\;{\bar{\theta }}_{6}=-diag\left({\bar{U}}_{i}\right),\;{\bar{\theta }}_{7}=-diag({\bar{Q}}_{i}),{\bar{\theta }}_{8}=-diag({\bar{U}}_{i}),\;\xi_{i}>0,\;i=1,\ldots ,\;k.$$

*Then, the optimal state-feedback gain is then calculated by* $K=YX_{1}^{-1}$ *where the augmented closed-loop matrix is denoted as* $A_{0}=A+BK$*.*

Proof See the appendix in the article's supplementary files. □

The primary importance of the presented robust approach is to provide a method to formulate the uncertainties caused by the cyber attacks instead of modeling with stochastic processes (system uncertainties and insufficient information from attackers are mostly modeled by considering noise and stochastic processes). Thus, we proposed a delay-independent $H_{\infty }$ approach to reduce the disturbances' effects caused by the attacker and increase the system's robustness. Consequently, finding a solution to the discussed optimization problem yields to providing a method to counteract the disturbing effects of the attacker. However, it is not straightforward to propose an analytical solution in general, and hence, the robust protocol is formulated using LMI methods. The developed robust strategy performs conservatively, and we tried to reach the optimal solution using the descriptor representation stated in (17) and (18).

In cases that it is hard to satisfy proposed LMI conditions, the main LMI problem can be reformulated to a convex optimization problem with LMI conditions to provide a trade-off between the controller's performance and the system's sensitivity to the disturbances by setting the LMI conditions less than a constant error value. On the other hand, the considered attack form in (4) is formulated in a relatively complex formation, and therefore, further equations are derived in general conditions. For exceptional cases, feasible solutions can be achieved.

Remark 1 If we assume that the attacker has complete access to the control network and can inject time delays into the communication channels (worst-case attack scenario), consequently, the immediate measurement of the process state x(t) and the actuator signal u(t)=Kx(t), will be replaced by $\overset{˜}{u}\left(t\right)=u\left(t-g_{0}\right)=Kx\left(t-g_{0}\right)$. Hence, the dynamic of the system (4) is reduced to

(24) $$\overset{˙}{x}\left(t\right)=\left(A+\Delta A\right)x\left(t\right)+B_{d}u(t-g_{0})+\sum_{i=1}^{k}{\overset{˜}{D}}_{i}\overset{˙}{x}\left(t-g_{i}(t)\right)+\sum_{i=2}^{k}H_{i}x\left(t-g_{i}(t)\right).$$

According to (24), by substituting $H_{1}$ by $B_{d}(YX_{1}^{-1})\;$ and then setting *B=0* in LMI condition (22), a time-independent criterion for analyzing the stability condition of the system (24) using the results in Theorem 1 will be obtained.

Remark 2 It should be noted that there is always a small amount of delay in transmitting data packets in smart network control systems, even without any attacks in the system. When time-delay attacks occur, the attacker's delay is augmented to this intrinsic network communication's delay value. Besides, in analyzing delays in small-scale networks, only the delay from the time-delay attack is mostly considered to derive formulas. However, in large-scale networks such as large-scale load frequency control networks with time-delay attacks [45], the system's delay should also be considered to avoid the network's instability in a large-scale fashion. In this article's formulations, the augmented delay term is assumed to be a general delay term to cover the system's delay, too. By this assumption, this paper's methodology can be implemented to overcome the issue of the existence of the system's delay and the attacker's delay together.

## 3 Fault detection for a robust, resilient control protocol against time-delay attacks

Knowing the attack event and the maximum tolerable time delay, an attack detector will direct the system into an alarm state. Under the investigated control strategy, the proposed scheme is maintained in a stable condition in the alert state by the designed robust controller. It remains in this state until the system status is restored. This method can be used as an economical and straightforward method to ensure industrial control systems' stability and safety. In the presented control method, due to the system being stable at the time of the attack event and due to the system states' limited values, we expect to be able to implement the residual-based fault detection methods or distance selection criteria to detect a fault in the system. In this section, we compare and analyze the ability of different attack detection techniques.

### 3.1 Fault detection based on maximum correlation

The correlation coefficient is one of the criteria used to determine the association between two variables. The correlation coefficient indicates the severity of the relationship and the type of connection (direct or inverse). This coefficient is between 1 and -1 and is zero if there is no relationship between the two variables. The correlation coefficient between the two input variables u(k) and the output of state x(k) is defined as follows:

(25) $$\rho \left(u\left(k\right),x\left(k\right)\right)=\frac{Cov\left(u\left(k\right),x\left(k\right)\right)}{\sqrt{D(u\left(k\right))}\sqrt{D(x\left(k\right))}}=\frac{\sum_{k=1}^{N}\left(u\left(k\right)-\mu_{u}\right)\left(x\left(k\right)-\mu_{x}\right)}{\sqrt{\sum_{k=1}^{N}{\left(u\left(k\right)-\mu_{u}\right)}^{2}}\sqrt{\sum_{k=1}^{N}{\left(x\left(k\right)-\mu_{x}\right)}^{2}}},$$

where $\mu_{x}$ and $\mu_{u}$ are the average of the input and output data. The most important thing to remember about the correlation coefficient is that the correlation coefficient only indicates the linear relationship between two variables.

In the non-attack mode, according to (4), the output change is statically related to the input change; that means any arbitrary inputs entirely generate different output values. So, if the data does not change, the output of the system will be retained. Thus, we expect that by injecting the attack's signal into the system, we will see a decrease in the correlation between the system's input and output variables. With a delay in the system, we expect the correlation coefficients to be decreased due to input and output sequences since the output does not correlate with the input. Therefore, in the delay-based attack detection algorithm and the general correlation-based delay estimation, the correlation coefficient between the input sequence and the output sequence can be calculated. Furthermore, the delay length corresponding to the maximum confidence coefficient can be considered as an estimation of the time delay value. Besides, the simulation results illustrate that the correlation coefficient is effective for accurately identifying the time-delay attack.

### 3.2 Fault detection based on K-L divergence analysis

One of the most common methods for detecting the malicious injected data is to monitor the dynamics of the measured data from the system and use the distance criterion. To quantify measurement changes, one can use both absolute distance indices and the K-L divergence criterion. For both distance indices, the two probability distributions *P* and *Q* must be considered, where the probability distribution *Q* is the statistical distribution of non-invasive measurements and *P* is also the statistical distribution of the measurement data exposed to injecting contaminated data. If there is no attack, the distance index will be relatively small, while the mentioned index will increase when the attacker's malicious data is injected into the system. By comparing the current time interval index *P* with *Q*, one can determine whether inaccurate data has been injected into the system or not. In the following, the common distance-based statistical attack detection criteria are reviewed and in the numerical results, their effectiveness in detecting the current article's proposed attack is investigated.

#### 3.2.1 Absolute distance criterion

A simple comparison criterion for the two probability distributions *P* and *Q* is the calculation of the absolute distance and can be defined as follows

(26) $$D_{A}\left(P∥Q\right)=\sum_{x}\left|P\left(x\right)-Q(x)\right|.$$

#### 3.2.2 K-L divergence measurement

For the two probability distributions *P* and *Q*, the K-L divergence criterion is defined as

(27) $$D_{KL}\left(P∥Q\right)=\sum_{x}P\left(x\right)\log P(x)/Q(x)\;.$$

Two essential features of the K-L divergence criterion are that it: (1) is always non-negative, i.e., $D_{KL}\left(P∥Q\right)\geq 0$; and (2) equals to zero if, and only if *P* = *Q*.

When the inaccurate, malicious data is injected into the system, the probability distribution of the altered measurement data deviates from the probability distribution of the error-free data, resulting in a more considerable K-L divergence calculated value.

Corollary 1 *If the two distributions P and Q are Gaussian, the introduced K-L divergence criterion can be simplified as the following*

(28) $$D_{KL}\left(P∥Q\right)=\frac{1}{2}\;\left(\;\log\frac{\left|\Sigma_{Q}\right|}{\left|\Sigma_{P}\right|}\;-n+tr\left(\Sigma_{Q}^{-1}\Sigma_{P}\right)\right)+\frac{1}{2}\;{\left(\mu_{Q}-\mu_{P}\right)}^{T}\Sigma_{Q}^{-1}(\mu_{Q-}\mu_{P}),$$

*where* $\mu_{P}$ *and* $\Sigma_{P}$ *are the mean and covariance of the sequence P, and also,* $\mu_{Q}$ *and* $\Sigma_{Q}$ *are defined as the mean and covariance of the sequence Q. Also, n is the dimension of the sequences in general.*

## 4 Numerical simulations

The effectiveness of the proposed robust $H_{\infty }$ control strategy under a time-delay attack is illustrated in this section.

An uncertain CPS with a malicious time-delay attack is assumed as the following form:

(29) $$\overset{˙}{x}\left(t\right)=\left(A+\Delta A\right)x\left(t\right)+Bu\left(t\right)+({\bar{D}}_{1}+\Delta {\bar{D}}_{1})\overset{˙}{x}\left(t-g(t)\right)+H_{1}x\left(t-g(t)\right),$$

(30) $$g(t)=T_{0}\left|\sin(3t)\right|,$$

where

$$A=\left[\begin{array}{ccc} -2\; & -0.1\; & 0\\ 0\; & -0.3\; & 0.5\\ 1\; & 0\; & -1 \end{array}\right],\;B=\left[\begin{array}{c} -1\\ 1.5\\ 1 \end{array}\right],\;{\bar{D}}_{1}=\left[\begin{array}{ccc} -0.2\; & -0.5\; & 0\\ 0\; & 0.3\; & 0\\ 1\; & 0\; & -0.6 \end{array}\right],H_{1}=\left[\begin{array}{ccc} -1\; & 0.1\; & 0\\ 0\; & 0.2\; & 0\\ 0\; & -1\; & 0.2 \end{array}\right],$$

and

$$\Delta A=\left[\begin{array}{ccc} 0.01\; & 0.1\; & -0.1\\ 0.04\; & 0.4\; & 0.4\\ 0\; & 0.01\; & 0.02 \end{array}\right],\Delta {\bar{D}}_{1}=\left[\begin{array}{ccc} -0.01\; & 0.1\; & 0.05\\ 0\; & 0.5\; & 0.05\\ 1\; & 0\; & -0.05 \end{array}\right]\;.$$

The total simulation time was set to *20 sec*, and the sampling time to *0.02 sec*. Based on the considered attack scenario, a time-delay attack is injected at time *t = 8 sec* with the maximum delay $T_{0}$ *= 4 sec* to all system states.

**Figure 2:**
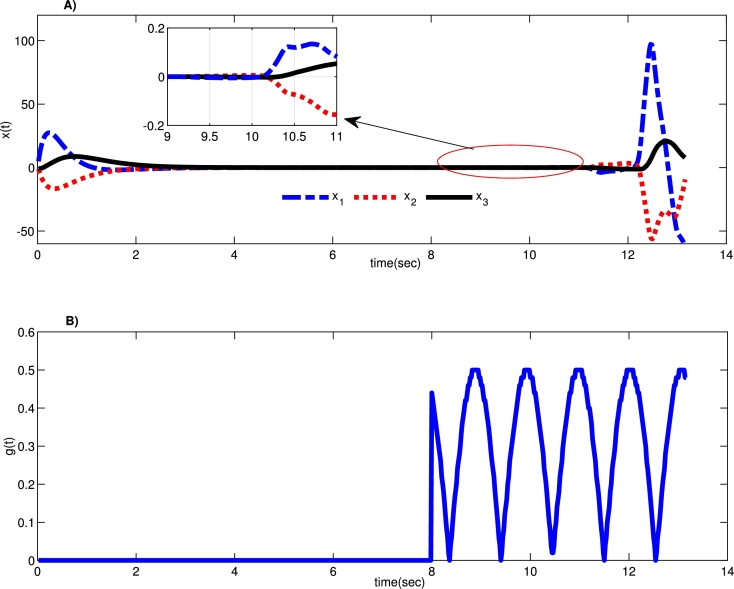
A) Instability of the uncertain system states trajectory by implementing conventional robust controller [4] under the time-delay attack, and B) assumed time delay attack *g*(*t*).

Fig. 2 shows the system's instability under this attack with the formerly developed robust controller as the one stated in [4]. We can conclude from Fig. 2 that designing a new robust control strategy is a must. Therefore, according to the developed robust control strategy, the feedback gain matrix *K* is calculated as

$$K=\;\left[\begin{array}{ccc} -0.0101\; & 0.164\; & -0.175 \end{array}\right].$$

**Figure 3:**
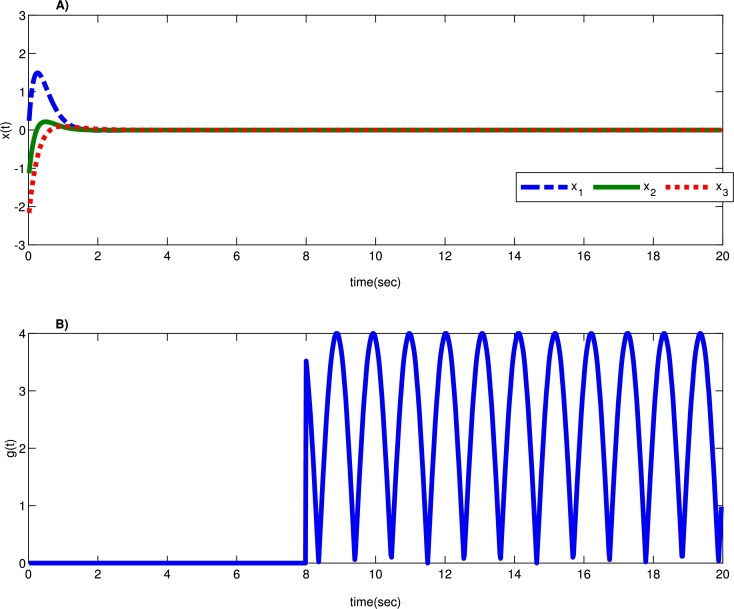
A) System states convergence against time-delay attack with the proposed robust controller, and B) assumed time delay attack *g*(*t*).

**Figure 4:**
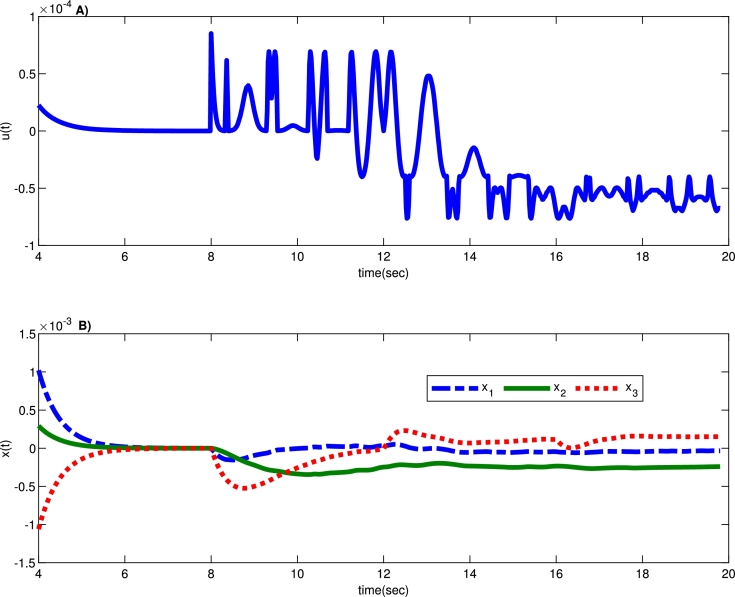
A) The proposed bounded robust control trajectory and B) system states trajectory under the time-delay attack injected at time *t* = 8 *sec*.

Moreover, Fig. 3 depicts the provided system's stability with the proposed robust controller against time-delay attacks. Additionally, Fig. 4 shows that system states and the proposed robust control signal under the time-delay attack remain bounded. As a result, the system could tolerate unknown time-delay attacks with the developed robust control strategy with the presented robust approach. Additionally, the system states' mean square error (MSE) values from the proposed robust control method based on various uncertainties under time-delay attacks are calculated in Table 1. According to Table 1, system states remain bounded under time-delay attacks with different uncertainty values.

**Table 1:** Calculated MSE of system states with the proposed robust control strategy against time-delay attacks under various system uncertainties.

${\left\|\Delta B\right\|}_{2}$	*MSE*(*x*_1_)	*MSE*(*x*_2_)	*MSE*(*x*_3_)	*sum*
0	0.0211	0.0148	0.0129	0.0489
0.4	0.0341	0.0156	0.0194	0.0692
0.8	0.0549	0.0165	0.0370	0.1085
1.2	0.0865	0.0177	0.0678	0.1720

**Figure 5:**
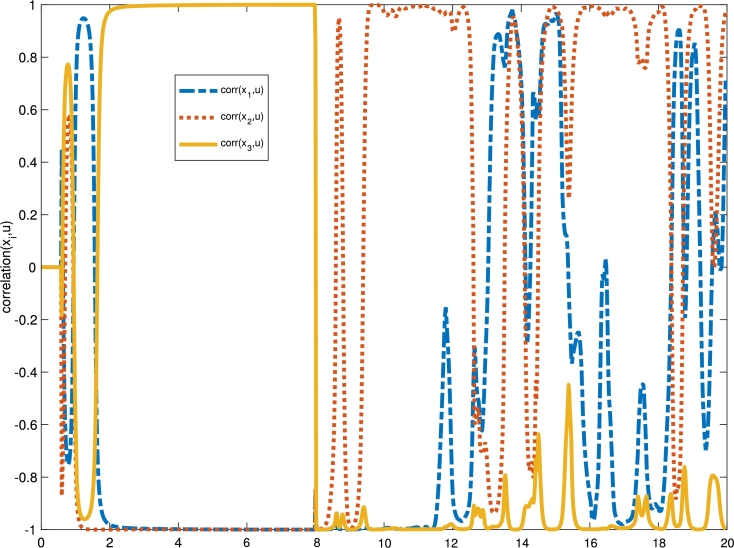
Correlation trajectory between states and control signal before and after the time-delay attack's existence.

Next step is to detect the attack's occurrence with various fault detection methods introduced in previous sections. Fig. 5 indicates the calculated correlation coefficient values before and after the time-delay attack. It can be observed that for data without attack, correlations are relatively close to 1 or -1, while the time-delay attack decreases the correlation coefficient value.

**Figure 6:**
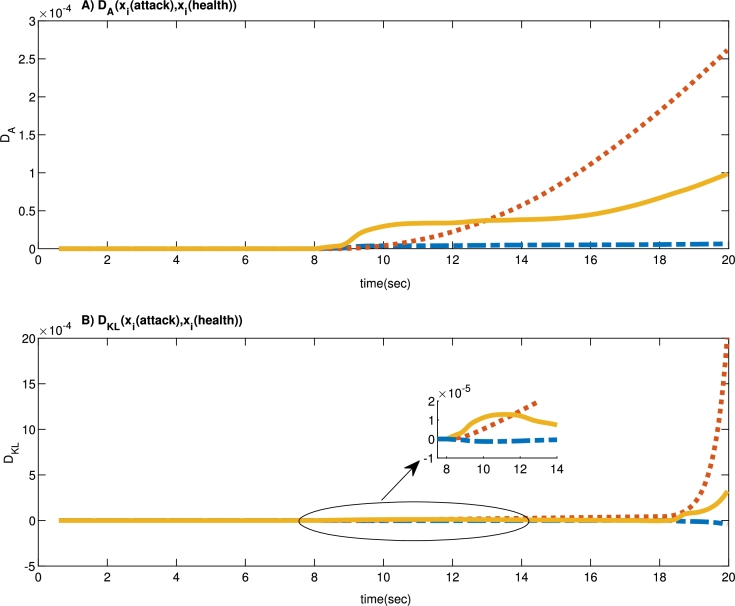
Measurement variation with false data injection attacks in *t* = 8 *sec*: A) Absolute distance value *D*_*A*_. B) K-L divergence value *D*_*KL*_.

Another way to detect faults can be developed by using the absolute distance criterion. Absolute distances $D_{A}$ and $D_{KL}$ between *P* (system with attack) and *Q* (system without attack) are indicated in Fig. 6. So, it can be inferred that injecting false data attacks to the system enhances the Absolute distance values $D_{A}$ and $D_{KL}$.

In false data injection attacks, compared with absolute distance (Fig. 6.a), the K-L distance (Fig. 6.b) is more significant. Therefore, in total, the absolute distance criterion is not an ideal candidate to test false data injection attacks' existence. To detect false data injection attacks with the K-L divergence criterion, we should analyze and set its detection threshold from the standard data. If the K-L divergence runtime is larger than the threshold, false data have likely been injected into the system.

It is worth noticing that the absolute distance and K-L divergence methods only require the system's output data in a non-attack mode or at least an accurate estimation of the system's output. Since the designed controller is relatively resistant to attack, the system's output data does not change much abruptly, and based on Fig. 6, there would be no significant changes in $D_{A}$ and $D_{KL}$ at the time of the onset of the attack. Although these attack detection parameters grow over time, injection of contaminated data into the system can be detected by decreasing the correlation level of the system's input and output data at the time of the attack event.

Remark 3 By the developed control method, the goal is to prevent the system from entering the destruction phase and, at the same time, to detect attacks. Therefore, previously introduced residual-based detection methods will be significantly ineffective in detecting attacks with low-amplitude or a small number of delays. So, considering a delay in the system with the variable sinusoidal behavior is practically the worst-case scenario. Based on this article's approach, the system can maintain its stability under this condition, as shown in simulation results. Thus, the system's response accuracy will be much higher than other control strategies, specifically in industrial systems.

Remark 4 Since the proposed robust control strategy can maintain the system states bounded under time-delay attacks with time-varying and unknown delays, the trustworthiness of the system's performance is increased. Moreover, by proposing the statistical methods to detect faults for the mentioned attacks, we illustrated in simulation results that attacks can be detected at the time of their occurrence and thus, we stated the term ‘Trust-based’ in the paper title to state that both provided fault detection and robust control strategies are trustworthy.

## 5 Conclusions

This article has proposed the secure, robust control design issue against unknown time-delay attacks of uncertain CPSs. The system's stability has been analyzed using a descriptor model representation and appropriate Lyapunov functional conditions for the closed-loop system. Furthermore, closed-loop security has been guaranteed by calculating the optimal feedback control gain in linear matrix inequalities (LMIs). Different time-delay attack detection frameworks have been proposed and compared according to the statistical analysis fault detection methods such as correlation analysis and Kullback-Leibler (K-L) divergence criteria from the mathematical view and in simulations. Finally, numerical results illustrated that the closed-loop uncertain system could remain stable with the proposed robust controller under time-delay attacks. The proposed approach can be applied to controlling systems with inaccurate models with variable environmental changes. Besides, suppose the system includes some delays or a threat of system instability due to delay effects caused by environmental changes. In that case, the presented conservative robust approach can be used to provide the system's stability, and it will enhance the system's resiliency. If the injected attack signals involve non-Gaussian distributions, new robust approaches should be developed since this paper's design cannot maintain the system's stability under non-Gaussian adversaries. Thus, subsequent studies include the security analysis of various types of CPSs in smart infrastructures under time-delay adversaries added to different types of Gaussian/non-Gaussian cyber threats and the design of associated fault detection and protection strategies.

## Declarations

### Author contribution statement

S. Baromand: Conceived and designed the experiments; Performed the experiments; Analyzed and interpreted the data; Contributed reagents, materials, analysis tools or data; Wrote the paper.

A. Zaman: Conceived and designed the experiments; Analyzed and interpreted the data; Contributed reagents, materials, analysis tools or data; Wrote the paper.

L. Mihaylova: Analyzed and interpreted the data; Contributed reagents, materials, analysis tools or data; Wrote the paper.

### Funding statement

This work was supported by ECO-Qube (956059).

### Data availability statement

Data will be made available on request.

### Declaration of interests statement

The authors declare no conflict of interest.

### Additional information

Supplementary content related to this article has been published online at https://doi.org/10.1016/j.heliyon.2021.e07294.

No additional information is available for this paper.

## Supplementary material

Appendix.pdf Appendix: Proof of Theorem 1. Appendix.pdf
