# Supplementary material for: Trust-based fault detection and robust fault-tolerant control of uncertain cyber-physical systems against time-delay injection attacks
Source: Heliyon. 2021 Jun 15;7(6):e07294. doi: 10.1016/j.heliyon.2021.e07294 (PMC8220189; doi:10.1016/j.heliyon.2021.e07294)
Supplement: Appendix.pdf — Appendix: Proof of Theorem 1. [file mmc1.pdf]

# Trust-Based Fault Detection and Robust Fault-Tolerant Control of Uncertain Cyber-Physical Systems Against Time-Delay Injection Attacks*

Salman Baromand[a], Amirreza Zaman[b], Lyudmila Mihaylova[c]

[a]*Department of Electrical Engineering,Fasa University, Fasa, Iran*
[b]*Control Engineering Group, Department of Computer Science, Electrical and Space Engineering, Luleå University of Technology, Luleå, Sweden*
[c]*Department of Automatic Control and Systems Engineering,University of Sheffield,Sheffield, UK*

## 1. Appendix: Proof of Theorem 1

To evaluate the proposed system's stability, the derivation matrix $\frac{dV}{dt}$ is obtained as follows

$$\frac{dV}{dt}=\zeta\begin{bmatrix} \widetilde{\psi}_1 & P^T\begin{bmatrix}0\\ \tilde{D}_1\end{bmatrix} & \dots & P^T\begin{bmatrix}0\\ \tilde{D}_k\end{bmatrix} & P^T\begin{bmatrix}0\\ H_1\end{bmatrix} & \dots & P^T\begin{bmatrix}0\\ H_k\end{bmatrix} \\ \begin{bmatrix}0 & \tilde{D}_1^T\end{bmatrix}P & -Q_1 & ... & 0 & 0 & \dots & 0 \\ \vdots & 0 & \ddots & 0 & 0 & \dots & . \\ \begin{bmatrix}0 & \tilde{D}_k^T\end{bmatrix}P & 0 & ... & -Q_k & 0 & \dots & 0 \\ \begin{bmatrix}0 & H_1^T\end{bmatrix}P & 0 & \dots & 0 & -U_1 & 0 & 0 \\ \vdots & \vdots & \dots & \vdots & 0 & \ddots & 0 \\ \begin{bmatrix}0 & H_k^T\end{bmatrix}P & 0 & ... & 0 & 0 & \dots & -U_k \end{bmatrix}\zeta^T. \tag{A1}$$

where

$$\zeta=\begin{bmatrix} x^T(t) & y^T(t) & y^T(t-g_1) & \dots & y^T(t-g_k) & x^T(t-g_1) & \dots & x^T(t-g_k)\end{bmatrix},$$

$$\widetilde{\psi}_1=P^T\begin{bmatrix}0 & I\\ \tilde{A}_0 & -I\end{bmatrix}+\begin{bmatrix}0 & \tilde{A}_0^T\\ I & -I\end{bmatrix}P+\begin{bmatrix}\sum_{i=1}^k U_i & 0\\ 0 & \sum_{i=1}^k Q_i\end{bmatrix}, \tag{A2}$$

Further, using Lemma 1, 2, for $\widetilde{\psi}_1$ we have:

$$\widetilde{\psi}_1=P^T\begin{bmatrix}0 & I\\ \tilde{A}_0 & -I\end{bmatrix}+\begin{bmatrix}0 & \tilde{A}_0^T\\ I & -I\end{bmatrix}P+\begin{bmatrix}\sum_{i=1}^k U_i & 0\\ 0 & \sum_{i=1}^k Q_i\end{bmatrix}\le\psi_1=P^T\begin{bmatrix}0 & I\\ A_0 & -I\end{bmatrix}$$
$$+\begin{bmatrix}0 & A_0^T\\ I & -I\end{bmatrix}P+\begin{bmatrix}\sum_{i=1}^k U_i & 0\\ 0 & \sum_{i=1}^k Q_i\end{bmatrix}+P^T\begin{bmatrix}0 & 0\\ 0 & \sum_{i=0}^m \xi_i^{-1}D_iD_i^T\end{bmatrix}P+\begin{bmatrix}\sum_{i=0}^m \xi_i E_i^T E_i & 0\\ 0 & 0\end{bmatrix}, \tag{A3}$$

*Amirreza Zaman(Corresponding author)Email: amirreza.zaman@ltu.se

* Funding received from the Horizon 2020 Research Programme of the European Commission under the grant number 956059 (ECO-Qube) is hereby gratefully acknowledged.

Consequently from (A1) - (A3), for the time derivative of $V(x,\ t)$, we have

$$\frac{dV}{dt} \leq \zeta \begin{bmatrix} \psi_1 & P^T \begin{bmatrix} 0 \\ \tilde{D}_1 \end{bmatrix} & \cdots & P^T \begin{bmatrix} 0 \\ \tilde{D}_k \end{bmatrix} & P^T \begin{bmatrix} 0 \\ H_1 \end{bmatrix} & \cdots & P^T \begin{bmatrix} 0 \\ H_k \end{bmatrix} \\ \begin{bmatrix} 0 & \tilde{D}_1^T \end{bmatrix} P & -Q_1 & 0 & 0 & 0 & \cdots & 0 \\ \vdots & 0 & \ddots & 0 & 0 & \cdots & 0 \\ \begin{bmatrix} 0 & \tilde{D}_k^T \end{bmatrix} P & 0 & 0 & -Q_k & 0 & \cdots & 0 \\ \begin{bmatrix} 0 & H_1^T \end{bmatrix} P & 0 & \cdots & 0 & -U_1 & 0 & 0 \\ \vdots & \vdots & \vdots & 0 & 0 & \ddots & 0 \\ \begin{bmatrix} 0 & H_k^T \end{bmatrix} P & 0 & 0 & 0 & 0 & 0 & -U_k \end{bmatrix} \zeta^T \tag{A4}$$

Further in (A4), we conclude:

$$\begin{bmatrix} \psi_1 & P^T \begin{bmatrix} 0 \\ \tilde{D}_1 \end{bmatrix} & \cdots & P^T \begin{bmatrix} 0 \\ \tilde{D}_k \end{bmatrix} & P^T \begin{bmatrix} 0 \\ H_1 \end{bmatrix} & \cdots & P^T \begin{bmatrix} 0 \\ H_k \end{bmatrix} \\ \begin{bmatrix} 0 & \tilde{D}_1^T \end{bmatrix} P & -Q_1 & \cdots & 0 & 0 & 0 & 0 \\ \vdots & 0 & \ddots & \vdots & \vdots & \cdots & \vdots \\ \begin{bmatrix} 0 & \tilde{D}_k^T \end{bmatrix} P & 0 & 0 & -Q_k & 0 & 0 & 0 \\ \begin{bmatrix} 0 & H_1^T \end{bmatrix} P & 0 & 0 & 0 & -U_1 & 0 & 0 \\ \vdots & \vdots & \vdots & \vdots & 0 & \ddots & 0 \\ \begin{bmatrix} 0 & H_k^T \end{bmatrix} P & 0 & 0 & 0 & 0 & 0 & -U_k \end{bmatrix}$$

$$= \begin{bmatrix} \psi_1 & P^T \begin{bmatrix} 0 \\ \overline{D}_1 \end{bmatrix} & \cdots & P^T \begin{bmatrix} 0 \\ \overline{D}_k \end{bmatrix} & P^T \begin{bmatrix} 0 \\ H_1 \end{bmatrix} & \cdots & P^T \begin{bmatrix} 0 \\ H_k \end{bmatrix} \\ \begin{bmatrix} 0 & \overline{D}_1^T \end{bmatrix} P & -Q_1 & 0 & 0 & 0 & 0 & 0 \\ \vdots & 0 & \ddots & 0 & \vdots & \cdots & \vdots \\ \begin{bmatrix} 0 & \overline{D}_k^T \end{bmatrix} P & \vdots & 0 & -Q_k & 0 & 0 & 0 \\ \begin{bmatrix} 0 & H_1^T \end{bmatrix} P & 0 & 0 & 0 & -U_1 & 0 & 0 \\ \vdots & \vdots & \cdots & \vdots & 0 & \ddots & 0 \\ \begin{bmatrix} 0 & H_k^T \end{bmatrix} P & 0 & ... & 0 & 0 & 0 & -U_k \end{bmatrix}$$

$$
+\begin{bmatrix}
0 & P^T\begin{bmatrix}0\\ \Delta\overline{D}_1\end{bmatrix} & \dots & P^T\begin{bmatrix}0\\ \Delta\overline{D}_k\end{bmatrix} & 0 & \dots & 0\\
\begin{bmatrix}0 & \Delta\overline{D}_1^T\end{bmatrix}P & 0 & \dots & 0 & 0 & 0 & 0\\
\vdots & \vdots & \dots & \vdots & \vdots & \vdots & \vdots\\
\begin{bmatrix}0 & \Delta\overline{D}_k^T\end{bmatrix}P & 0 & \dots & 0 & 0 & 0 & 0\\
0 & 0 & \dots & 0 & 0 & 0 & 0\\
\vdots & \vdots & \dots & \vdots & \vdots & \vdots & 0\\
0 & 0 & \dots & 0 & 0 & \dots & 0
\end{bmatrix}
$$

using Lemma 2, the above equation results in

$$
\begin{bmatrix}
0 & P^T\begin{bmatrix}0\\ \Delta\overline{D}_1\end{bmatrix} & \dots & P^T\begin{bmatrix}0\\ \Delta\overline{D}_k\end{bmatrix} & 0 & \dots & 0\\
\begin{bmatrix}0 & \Delta\overline{D}_1^T\end{bmatrix}P & 0 & \dots & 0 & 0 & 0 & 0\\
\vdots & \vdots & \dots & \vdots & \vdots & \vdots & \vdots\\
\begin{bmatrix}0 & \Delta\overline{D}_k^T\end{bmatrix}P & 0 & \dots & 0 & 0 & 0 & 0\\
0 & 0 & \dots & 0 & 0 & 0 & 0\\
\vdots & \vdots & \dots & \vdots & \vdots & \vdots & 0\\
0 & 0 & \dots & 0 & 0 & \dots & 0
\end{bmatrix}
$$

$$
\leq \begin{bmatrix}
P^T\begin{bmatrix}0 & 0\\ 0 & \sum_{i=1}^m \xi_i^{-1} D_i D_i^T\end{bmatrix}P & 0 & 0 & \dots & 0 & \dots & 0\\
0 & 2\xi_1\overline{\mathrm{E}}_1\overline{\mathrm{E}}_1^{\mathrm{T}} & 0 & \dots & 0 & \dots & 0\\
\vdots & \vdots & \ddots & \vdots & \vdots & & \vdots\\
0 & 0 & \dots & 2\xi_k\overline{\mathrm{E}}_{\mathrm{k}}\overline{\mathrm{E}}_{\mathrm{k}}^{\mathrm{T}} & 0 & \dots & 0\\
0 & 0 & 0 & 0 & 0 & \dots & 0\\
\vdots & \vdots & \vdots & \vdots & \vdots & \ddots & \vdots\\
0 & 0 & \dots & 0 & 0 & \dots & 0
\end{bmatrix} = \overline{\overline{\Gamma}}, \qquad (A5)
$$

Also according to the results of (A5), for (A4) can be concluded that:

$$
\frac{dV}{dt} \leq \zeta \begin{bmatrix}
\psi_1 & P^T\begin{bmatrix}0\\ \overline{D}_1\end{bmatrix} & \dots & P^T\begin{bmatrix}0\\ \overline{D}_k\end{bmatrix} & P^T\begin{bmatrix}0\\ H_1\end{bmatrix} & \dots & P^T\begin{bmatrix}0\\ H_k\end{bmatrix}\\
\begin{bmatrix}0 & \overline{D}_1^T\end{bmatrix}P & -Q_1 & \dots & 0 & 0 & \dots & 0\\
\vdots & 0 & \dots & 0 & \vdots & \dots & \vdots\\
\begin{bmatrix}0 & \overline{D}_k^T\end{bmatrix}P & 0 & \dots & -Q_k & 0 & \cdots & 0\\
\begin{bmatrix}0 & H_1^T\end{bmatrix}P & 0 & \dots & 0 & -U_1 & 0 & 0\\
\vdots & \vdots & \dots & \vdots & \vdots & \ddots & 0\\
\begin{bmatrix}0 & H_k^T\end{bmatrix}P & 0 & \dots & 0 & 0 & \dots & -U_k
\end{bmatrix}\zeta^T + \zeta\overline{\overline{\Gamma}}\zeta^T \qquad (A6)
$$

Thus by Schur complements, $\frac{dV}{dt} \leq 0$ if the given LMI holds:

$$W = \begin{bmatrix} \overline{\psi}_1 & \theta_1 & 0 & \theta_2 \\ * & \theta_9 & \theta_6 & 0 \\ * & * & \theta_7 & 0 \\ * & * & * & \theta_{10} \\ * & * & * & * \\ * & * & * & * \end{bmatrix} < 0 \tag{A7}$$

where

$$\overline{\psi}_1 = P^T A_0 + A_0^T P + \begin{bmatrix} \sum_{i=1}^k U_i & 0 \\ 0 & \sum_{i=1}^k Q_i \end{bmatrix} + P^T \begin{bmatrix} 0 & 0 \\ 0 & 2\sum_{i=1}^m \xi_i^{-1} D_i D_i^T \end{bmatrix} P$$

$$+P^T \begin{bmatrix} 0 & 0 \\ 0 & \xi_0^{-1} D_0 D_0^T \end{bmatrix} P + \begin{bmatrix} \sum_{i=0}^m \xi_i E_i^T E_i & 0 \\ 0 & 0 \end{bmatrix},$$

$$\theta_1 = vec\left\{P^T \begin{bmatrix} 0 \\ \overline{D}_i \end{bmatrix}\right\}^T,\ \theta_2 = vec\left\{P^T \begin{bmatrix} 0 \\ H_i \end{bmatrix}\right\}^T, \theta_6 = vec\left\{\overline{E}_i^T\right\}, \theta_7 = -diag\left(\xi_i^{-1}\ I\right),$$

$$\theta_9 = -diag(Q_i), \theta_{10} = -diag(U_i),\ i = 1, \ldots,\ k.$$

Then, from above LMI we have the following inequality:

$$W = \begin{bmatrix} \overline{\overline{\psi}}_1 & \theta_1 & 0 & \theta_2 & vec\{I\} & vec\{I\} \\ * & \theta_9 & \theta_6 & 0 & 0 & 0 \\ 0 & * & \theta_7 & 0 & 0 & 0 \\ * & * & * & \theta_{10} & 0 & 0 \\ * & 0 & 0 & 0 & \theta_{14} & 0 \\ * & 0 & 0 & 0 & 0 & \theta_{16} \end{bmatrix} < 0, \tag{A8}$$

where

$$\overline{\overline{\psi}}_1 = P^T \begin{bmatrix} 0 & I \\ A_0 & -I \end{bmatrix} + \begin{bmatrix} 0 & A_0^T \\ I & -I \end{bmatrix} P + P^T \begin{bmatrix} 0 & 0 \\ 0 & 2\sum_{i=1}^m \xi_i^{-1} D_i D_i^T \end{bmatrix} P$$

$$+P^T \begin{bmatrix} 0 & 0 \\ 0 & \xi_0^{-1} D_0 D_0^T \end{bmatrix} P + \begin{bmatrix} \sum_{i=0}^m \xi_i E_i^T E_i & 0 \\ 0 & 0 \end{bmatrix},$$

$$\theta_{14} = -diag(Q_i^{-1}), \theta_{16} = -diag(U_i^{-1}),\ \xi_i > 0,\ i = 1, \ldots, k.$$

by pre and post multiplying (A8) by $\Delta_2 = diag\left\{X,\ diag\left(Q_i^{-1}\right), I,\ diag(U_i^{-1}), I, I\right\}$ and $\Delta_2^T$ respectively, where $X = P^{-1}$, also denote $KX_1$ by $Y$ and $\overline{Q}_i = Q_i^{-1}$, $\overline{U}_i = U_i^{-1}$, and applying the Schur formula, we obtain the inequality shown in (22) in the article. This implies asymptotic stability of (4) for continuous functions and under Assumption A1, for continuously differentiable functions. ■
